# Molecular and entomological surveillance of malaria vectors in urban and rural communities of Benguela Province, Angola

**DOI:** 10.1186/s13071-024-06214-8

**Published:** 2024-03-06

**Authors:** Gonçalo Alves, Arlete Dina Troco, Gonçalo Seixas, Rebecca Pabst, Alfredo Francisco, Cani Pedro, Luzala Garcia, José Franco Martins, Sergio Lopes

**Affiliations:** 1 The Mentor Initiative, Burns House, Harlands Road, Haywards Heath, RH16 1PG UK; 2 Global Health and Tropical Medicine, GHTM, Associate Laboratory in Translation and Innovation Towards Global Health, LA-REAL, Instituto de Higiene e Medicina Tropical, Universidade Nova de Lisboa, UNL, Lisbon, Portugal; 3 World Vision International, Luanda, Angola; 4 National Malaria Control Programme, Ministry of Health, Luanda, Angola

**Keywords:** Angola, *Anopheles gambiae*, *Anopheles funestus*, *Anopheles vaneedeni*, Insecticide resistance, *Kdr* mutations

## Abstract

**Background:**

Malaria is a major public health problem in Angola, with *Anopheles gambiae* sensu lato (s.l.) and *An. funestus* s.l. being the primary vectors. This study aimed to clarify the information gaps concerning local *Anopheles* mosquito populations. Our objectives were to assess their abundance, geographical dispersion, and blood-feeding patterns. We also investigated their insecticide resistance. Molecular methods were used to identify sibling species, determine the origin of blood meals, measure *Plasmodium falciparum* infection rates, and detect the presence of knockdown resistance (kdr) mutations.

**Methods:**

Adult mosquitoes were collected indoors using CDC light traps from nine randomly selected households at two sentinel sites with distinct ecological characteristics. The samples were collected from 1 February to 30 June 2022. *Anopheles* mosquitoes were morphologically identified and subjected to molecular identification. Unfed *Anopheles* females were tested for the presence of *P. falciparum* DNA in head and thorax, and engorged females were screened for the source of the blood meals. Additionally, members of *An. gambiae* complex were genotyped for the presence of the L1014F and L1014S kdr mutations.

**Results:**

In total, 2226 adult mosquitoes were collected, including 733 *Anopheles* females. Molecular identification revealed the presence of *Anopheles coluzzii*, *An. gambiae* senso stricto (s.s.), *An. arabiensis*, and *An. funestus* s.s. Notably, there was the first record of *An. coluzzii*/*An. gambiae* s.s. hybrid and *An. vaneedeni* in Benguela Province. *Plasmodium falciparum* infection rates for *An. coluzzii* at the urban sentinel site and *An. funestus* s.s. at the rural site were 23.1% and 5.7%, respectively. The L1014F kdr mutation was discovered in both resistant and susceptible *An. coluzzii* mosquitoes, while the L1014S mutation was detected in *An. gambiae* s.s. for the first time in Benguela Province. No kdr mutations were found in *An. arabiensis.*

**Conclusions:**

This study provides valuable insights into the molecular characteristics of malaria vectors from the province of Benguela, emphasising the need for continuous surveillance of local *Anopheles* populations regarding the establishment of both kdr mutations for tailoring vector control interventions.

**Graphical abstract:**

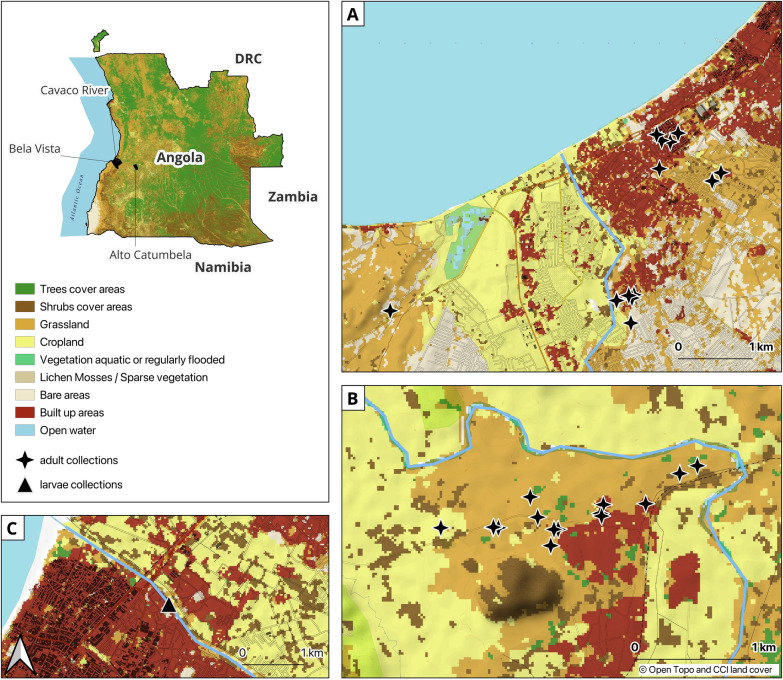

## Background

Malaria remains a major public health challenge, with an estimated 249 million cases and 608,000 deaths worldwide in 2022 [1]. In sub-Saharan Africa, the primary vectors responsible for malaria transmission are *Anopheles gambiae* senso stricto (s.s.), *An. coluzzii*, *An. arabiensis*, and *An. funestus* s.s. [2]. In Angola, malaria is endemic and mostly caused by *Plasmodium falciparum* [3]. Additionally, cases caused by *Plasmodium malariae* have been recorded in the province of Benguela [4]. In Angola, malaria transmission is complex with vectors occupying different eco-epidemiological areas. *Anopheles gambiae* s.s., *An. coluzzii*, *An. arabiensis*, and *An. funestus* are considered the primary vectors in Angola [3, 5–7]. Insecticide-based vector control strategies, such as the use of insecticide-treated nets (ITN) and indoor residual spraying (IRS), have significantly contributed to the reduction of the malaria burden [8]. However, the emergence and spread of insecticide resistance in these main vectors [9, 10] are threating the effectiveness of these interventions. Two main types of mechanism have been identified to be involved in insecticide resistance: target-site resistance, which involves mutations in the target proteins of insecticides, and metabolic resistance, which involves increased detoxification of insecticides [11]. One well-known target site resistance is the knockdown resistance (kdr) caused by mutations in the voltage-gated sodium channel gene, which compromises its binding to pyrethroid insecticides [12]. The identification of *Anopheles* species and their susceptibility statute to insecticides are essential for effective planning of vector control strategies. Molecular techniques have improved the accuracy of species identification within the *An. gambiae* complex and *Anopheles funestus* group [13, 14]. Furthermore, molecular methods, such as polymerase chain reaction (PCR) assays, have been developed to detect kdr mutations, enabling the assessment of the frequency and distribution of these resistance-associated mutations in field populations [15]. Despite the crucial role of vector control in reducing malaria transmission, little published evidence on Angola malaria vector abundance, behaviour, and insecticide susceptibility has been published in the past 20 years [16–20]. This study aimed to describe the local populations of *Anopheles* mosquito species in two districts of Benguela, with a focus on characterising the vector abundance, behaviour, and insecticide susceptibility of these populations. Using molecular methods to investigate the presence of knockdown resistance (kdr), *P. falciparum* infectivity rates, and blood meal sources, the outcomes will aid in bridging knowledge gaps concerning malaria vectors in Benguela Province.

## Methods

### Study areas

**Fig. 1:**
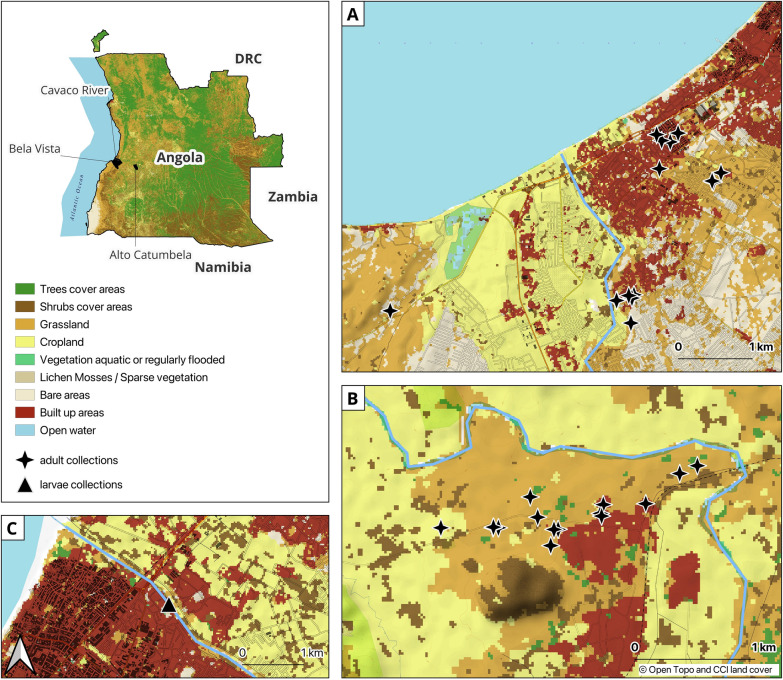
Mosquito collection sites. **A** Adult collection site at Bela Vista in Benguela District. **B** Adult collection sites at Alto Catumbela in Ganda District. **C** Larva collection site at Cavaco River at Benguela District

The study was conducted from 1 February to 30 June 2022 at two sentinel sites in the province of Benguela. Bela Vista (Fig. 1A, urban site, 12°37′27.8ʺS, 13°22′23.4ʺE) is an urban neighbourhood located in the city of Benguela at an average altitude of 20 m. The climate is characterised by a dry season (May to September) and a rainy season that extends from October to April with an average rainfall of 12.9 mm (October) to 33.9 mm (March). The average temperature varies from 30 °C (March/April) to 19 °C (July/August). Cavaco River (Fig. 1C, 12°34′10.0ʺS 13°25′11.5ʺE) is located at the urban north border of the city of Benguela. It is used by the local population during the wet and dry season to collect water for different uses. During the dry season, the local population dig wells in the dry riverbed to facilitate water collection. Alto Catumbela in the district of Ganda (Fig. 1B, rural site, 12°56′15.3ʺS 14°45′19.1ʺE) is a rural community located 210 km east of Bela Vista at an altitude of 1244 m. The predominant activity in this area is agriculture and cattle breeding. Families who own cattle keep them in corrals or fenced off near their houses. Temperatures range from 31 °C (September) to 10 °C (June/July). The rainy season lasts from October to April, with a monthly rainfall average of 37 mm in April to 130.6 mm in December. In both locations, during field work, we observed that households use ITN, aerosol insecticides, and mosquito-repelling coils to protect themselves against mosquito bites.

### Adult sampling methods

Adult mosquitoes were collected indoors from the Bela Vista and Alto Catumbela sentinel sites. At each sentinel site, nine houses were randomly selected at least 50 m apart. The selected houses had cement or mud walls, painted or unpainted, with or without ceilings, and roofs made of sheet metal or traditional materials. CDC light traps (CDC-LT; Model 512; John W. Hock Company, Gainesville, FL, USA) were used overnight to collect mosquitoes for 3 consecutive nights in three different houses each night. The CDC-LTs were installed circa 150 cm above the ground at the foot end of the sleeping area where people were sleeping under a mosquito net. Mosquitoes were captured between 18:00 and 07:00. Non-anopheline species were discarded after counting. In households where permission was granted for the collection of adult mosquitoes indoors, the collection procedure was communicated to the household head, who provided their consent.

### Larva collections and WHO susceptibility test

To test the insecticide susceptibility of mosquitoes, the WHO tube test was performed using papers impregnated with alpha-cypermethrin at a diagnostic concentration of 0.05% [21]. The tests were performed using F0 generation, 2–5-day-old females collected as larvae in June 2022 from shallow wells in the dry riverbed of Cavaco River (Fig. 1C, 12°34′10.0ʺS 13°25′11.5ʺE). Collected larvae were brought to the field insectary in Benguela and reared to adult stage. Before the start of the test, a preliminary morphological identification was carried out on live adult *Anopheles* mosquitoes. Mosquitoes identified as *Anopheles gambiae* s.l. were sorted for WHO tube tests. At the end of the test, a confirmatory morphological identification was carried out to ensure that only *Anopheles gambiae* s.l. were included in the mortality analysis. Any other mosquito species found were excluded from the final count. The population was classified as “resistant” if mortality < 90%, “possible resistant” if mortality < 98%, and “susceptible” if mortality ≥ 98%. After the assay, mosquitoes were individually stored in labelled microtubes containing silica gel for further molecular processing.

### Sample processing

#### *Anopheles* mosquito species identification

Prior to DNA extraction, female *Anopheles* mosquitoes were identified to species using morphological keys [22, 23]. Morphological identification was performed by trained entomologists. Abdomens from captured female *Anopheles* mosquitoes were categorised as unfed or fed. Samples were stored individually in labelled microtubes containing silica gel for molecular analysis at room temperature. Genomic DNA was extracted according to Collins et al. [24]. DNA was used for identification of sibling species, the presence of *P. falciparum*, blood meal source, and presence of kdr mutations. Molecular sibling species identification was conducted on randomly selected morphological identified members of the *An. gambiae* s.l. and *An. funestus* s.l. For *An. gambiae* s.l., molecular identification was conducted by targeting species-specific polymorphisms at the intergenic spacer (IGS) of the ribosomal DNA [25] and by a PCR assay targeting the SINE 200X6.1 retrotransposon insertion [26]. Specimens were identified as *An. coluzzii*, *An. gambiae* s.s., *An. coluzzii*/*An. gambiae* s.s. hybrids, *Anopheles melas*, or *An. arabiensis* if they had coincident species-specific patterns for both markers. For the *An. funestus* s.l. members, which include *An. funestus* s.s., *Anopheles parensis*, *An. rivulorum*, *An. leesoni*, *An. rivulorum*-like, and *An. vaneedeni*, molecular identification was conducted using the protocol described by Koekemoer et al. [14]. All PCR assays contained negative controls (no DNA template) and positive controls, consisting of samples of known specimen molecular identification. *Anopheles gambiae* s.l. and *An. funestus* s.l. PCR species-specific identifications giving a negative result and randomly selected specimens of less common species of *Anopheles* mosquitoes were submitted to Cytochrome Oxidase I (COI) barcode sequencing for species identification, following Folmer et al. [27]. Comparison between morphological and molecular identification was performed to assess the accuracy of the morphological identification. All COI sequences have been submitted to NCBI and are available online under GenBank accession numbers OR839824–OR839854.

#### Identification of the blood meal source

The origin of blood meals in blood-fed *Anopheles* mosquitoes was performed using a multiplex PCR [28], targeting human, cow, dog, goat, and pig DNA. Positive and negative controls were included in all reactions.

#### Detection of *Plasmodium falciparum* DNA in *Anopheles* mosquitoes

Molecular procedure describe by Demas et al. [29] was used to detect the presence of *P. falciparum* DNA. Since this technique does not allow to distinguish between infected and infectious mosquitoes, only heads and thoraxes of unfed mosquitoes were used. Preference was given to mosquitoes molecularly identified as *An. gambiae* s.s., *An. coluzzii*, *An. arabiensis*, and *An. funestus* s.s.

### Kdr mutation detection

Kdr mutations were surveyed in *An. gambiae* s.l. exposed and not exposed to insecticides, following the well-established protocols targeting kdr-West (L1014F) and kdr-East (L1014S) mutations [12, 15].

### Data analysis

The accuracy of morphological identification was determined as the ratio between molecular species confirmations and the total number of morphological identifications subject to molecular identification by species. Human blood index (HBI) was calculated as the number of mosquitoes fed on humans (including mixed blood meal origins) by the total number of blood-fed anopheline mosquitoes analysed. Infection rate (IR%) was estimated as the proportion of unfed *Anopheles* females positive for *P. falciparum* DNA. To assess the association between kdr genotypes and resistance phenotypes, in *An. gambiae* complex, Fisher’s exact test was calculated with contingency tables using GraphPad Prism (version 8.0.1).

## Results

### *Anopheles* mosquito species composition

From February to June 2022, 2226 adult mosquitoes were collected using indoor CDC-LT. Among the collected mosquitoes, 748 (33.6%; 733 females and 15 males) were identified as anopheline mosquitoes and 1478 (66.4%; 1148 females and 330 males) were classified as non-anopheline mosquitoes. Out of the total *Anopheles* females collected as adults, 622 (84.9%) were morphologically identified to species or complex of species, while 111 (15.1%) could not be identified because of the absence of anatomical parts. Of the larvae collected from Cavaco River and reared in an insectary to an adult stage, 60 were identified as *An. gambiae* s.l. The district of Ganda contributed 89.9% (*n* = 713) while Benguela District contributed 10.1% (*n* = 80) of the female *Anopheles* mosquitoes collected and morphologically identified (Table 1). A subsample of 155 *An. gambiae* s.l. and 375 *An. funestus* s.l. were molecularly processed for species identification. The molecular identification of the 155 *An. gambiae* s.l. indicated the presence of *An. arabiensis* (*n* = 26), *An. coluzzii* (*n* = 75), *An. gambiae* s.s. (*n* = 49), and *An. coluzzii/An. gambiae* s.s. hybrid (*n* = 2); three showed no amplification. From the 375 *An. funestus* s.l. mosquitos, 360 were identified as *An. funestus* s.s. and two as *An. vaneedeni*, and 13 specimens showed no amplification (Table 2). From those that did not show amplification on species-specific molecular identification (*n* = 16), five *An. funestus* s.l. and two *An. gambiae* s.l., as well as 34 anophelines of other *Anopheles* species, were identified by COI barcode analysis (Table 3). The accuracy of the morphological identification was determined by crossing morphological and molecular identification. This reveled an overall accuracy of 94.5% (533/504). *Anopheles pretoriensis*, *An. coustani* s.l., and *An. maculipalpis* had 100% accuracy, followed by *An. gambiae* s.l. (98.1%), *An. funestus* s.l. (97.3%), and *An. squamosus* (85.7%). Morphological identifications failed on *An. obscurus*, *An. rhodesiensis* s.l., *An. ruarinus*, *An. argenteolobatus*, and *An. tenebrosus* (Table 4). Molecular species identification revealed a total of 11 species, including the hybrids (Table 5). Overall, more adult *Anopheles* mosquitoes were caught in the rainy season (*n* = 388) than in the dry season (*n* = 164) (Table 5). Overall, in the dry season, the predominant species collected was *An. funestus* s.s. followed by *An. coluzzii, An. arabiensis*. and *An. gambiae* s.s. (Table 5). In the rainy season, *An. funestus* s.s. was the predominant species recorded followed by *An. gambiae* s.s., *An. coluzzii*, and *An. arabiensis*. In the rainy season, *An. squamosus* was also registered in relatively high numbers (*n* = 11; 2.3%) (Table 5). The two specimens of the hybrid *An. coluzzii*/*An. gambiae* s.s. were registered in the dry season, in both Cavaco River and Alto Catumbela collection sites, the former as a larva and the latter as an adult (Table 5). In terms of geographical distribution, there were clear differences, with *An. coluzzii* being found in Bela Vista and Cavaco River sentinel sites (Table 5).

**Table 1:** Results of female *Anopheles* spp. morphological identification

Morphological identification to species	*N*	Sentinel sites		
		District of Benguela		District of Ganda
		Bela Vista	Cavaco River^a^	Alto Catumbela
*An. funestus* s.l.	430	1	–	429
*An. gambiae* s.l.	160^b^	16	60	84
*An. squamosus*	39	0	–	39
*An. coustani* s.l.	20	0	–	20
*An. obscurus*	8	0	–	8
*An. azevedoi*	6	2	–	4
*An. ruarinus*	5	0	–	5
*An. rhodesiensis *s.l.	4	0	–	4
*An. pretoriensis*	2	0	–	2
*An. azaneae*	1	0	–	1
*An. argenteolobatus*	1	0	–	1
*An. caroni*	1	0	–	1
*An. jebudensis*	1	0	–	1
*An. maculipalpis*	1	0	–	1
*An. salbai*	1	0	–	1
*An. tenebrosus*	1	0	–	1
*An. turkudi*	1	0	–	1
*An. *spp.	111	1	–	110
Total n, (%)	793	20 (2.5)	60 (7.6)	713 (89.9)

^a^Only larval collections. ^b^Include mosquitoes collected as larvae and reared to adult stage

**Table 2:** Species-specific molecular identification of *Anopheles gambiae* s.l. and *An. funestus* s.l.

Morphological identification	*N*	PCR species-specific identification	Benguela District		Ganda District	Total
Species identified		Species identified	Bela Vista	Cavaco River ^b^	Alto Catumbela	
*An. gambiae* s.l.	155	*An. arabiensis*	0		26	26
		*An. coluzzii*^a^	16	59	0	75
		*An. gambiae* s.s	0		49	49
		*An. coluzzii*/*An. gambiae* s.s. hybrid	0	1	1	2
		No amplification	0		3	3
		Total processed	16	60	79	155
*An. funestus *s.l.	375	*An. funestus* s.s.	1		359	360
		*An. vaneedeni*	0		2	2
		No amplification	0		13	13
		Total processed	1		374	375

^a^Includes mosquitoes collected as larvae and adults. ^b^Only larval collections

**Table 3:** COI barcoding analysis of *Anopheles* mosquito

Species identified by morphological identification	*N*	COI barcoding species identification	Alto Catumbela	Total
*An. squamosus*	14	*An. squamosus*	12	12
		No result	2	2
*An. obscurus*	8	*An. funestus *s.s.	7	7
		*An. vaneedeni*	1	1
*An. funestus* s.l	5	*An. arabiensis*	1	1
		*An. funestus *s.s.	3	3
		*An. azevedoi*	1	1
*An. rhodesiensis* s.l	4	*An. gambiae* s.s.	1	1
		*An. funestus *s.s.	3	3
*An. gambiae* s.l	2	*An. funestus *s.s.	2	2
*An. pretoriensis*	2	*An. pretoriensis*	2	2
*An. ruarinus*	2	*An. funestus *s.s.	2	2
*An. argenteolobatus*	1	No result	1	1
*An. coustani *s.l.	1	*An. coustani*	1	1
*An. maculipalpis*	1	*An. maculipalpis*	1	1
*An. tenebrosus*	1	*An. coustani*	1	1

**Table 4:** Accuracy of morphological identification

Morphological identification		*Molecular identification, n*											No result	Accuracy identification (%)
Species	N	*An. gambiae s.s*	*An. coluzzii*	*An. arabiensis*	*Hybrid*	*An. funestus* s.s	*An. vaneedeni*	*An. azevedoi*	*An. coustani*	*An. maculipalpis*	*An. pretoriensis*	*An. squamosus*		
*An. funestus* s.l.	375	–	–	1	–	363	2	1	–	–	–	–	8	97.3
*An. gambiae* s.l.	155^a^	49	75^a^	26	2^a^	2	–	–	–	–	–	–	1	98.1
*An. squamosus*	14	–	–	–	–	–	–	–	–	–	–	12	2	85.7
*An. obscurus*	8	–	–	–	–	7	1^b^	–	–	–	–	–	–	0.0
*An. rhodesiensis* s.l.	4	1	–	–	–	3	–	–	–	–	–	–	–	0.0
*An. pretoriensis*	2	–	–	–	–	–	–	–	–	–	2	–	–	100.0
*An. ruarinus*	2	–	–	–	–	2	–	–	–	–	–	–	–	0.0
*An. argenteolobatus*	1	–	–	–	–	–	–	–	–	–	–	–	1	0.0
*An. coustani s.l.*	1	–	–	–	–	–	–	–	1^c^	–	–	–	–	100.0
*An. maculipalpis*	1	–	–	–	–	–	–	–	–	1	–	–	–	100.0
*An. tenebrosus*	1	–	–	–	–	–	–	–	1	–	–	–	–	0.0
Total	564	50	75	27	2	377	3	1	2	1	2	12	12	94.5

^a^Includes mosquitoes collected as larvae and adults. ^b^*Anopheles vaneedeni* or *An. longipalpis*. ^c^*Anopheles coustani* or *An. ziemanni*

**Table 5:** Seasonal and geographical distribution *Anopheles* mosquito in Benguela Province

Variables	*An. gambiae s.s*	*An. coluzzii*	*An. arabiensis*	*Hybrid*	*An. funestus s.s*	*An. vaneedeni*	*An. azevedoi*	*An. coustani*	*An. maculipalpis*	*An. pretoriensis*	*An. squamosus*	Total, n (%)	Nr. of species
Total	50	75	27	2	377	3	1	2	1	2	12	552 (100.0)	11
Season													
Dry (May to June)	10	60^a^	23	2	66	–	1	1	-	-	1	164 (29.7)	8
Rainy (February to April)	40	15	4	–	311	3	-	1	1	2	11	388 (70.3)	9
Sentinel site													
Benguela District													
Bela Vista	–	16	–	–	1	–	–	–	–	–	–	17 (3.1)	2
Cavaco River	–	59^a^	–	1^a^								60 (10.9)	1
Ganda District													
Alto Catumbela	50	–	27	1	376	3	1	2	1	2	12	475 (86.0)	10

^a^Adults collected as larvae

### WHO susceptibility test

In the insecticide susceptibility assays conducted on *An. gambiae* s.l. from Benguela using 0.05% alpha-cypermethrin, a mortality rate of 57.6% was observed. Notably, only 60 *An. gambiae* s.l. females were exposed to impregnated filter papers. This sample size was not considered robust according to WHO standards. Nonetheless, these preliminary results suggested that this population may exhibit phenotypic resistance to alpha-cypermethrin. Molecular identification revealed that this population was *An. coluzzii* (*n* = 59) and *An. coluzzii/An. gambiae* s.s. (*n* = 1) (Table 2).

### Origin of *Anopheles* blood meals

The origin of blood meals of *Anopheles* mosquitoes collected indoors using CDC-LT showed that humans were the main host (71%) in both sites (Table 6). In Bela Vista, *An. coluzzii* was the only species screened with a human blood index (HBI) of 0.67. In Alto Catumbela, where four species were tested, the highest HBI was registered for *An. funestus* s.s. (0.84) followed by *An. vaneedeni* and *An. arabiensis* (both with 0.50) and *An. gambiae* s.s. (0.25). *Anopheles arabiensis*, *An. funestus*, and, *An. vaneedeni* collected indoors had also fed on cows.

**Table 6:** Origin of the blood meal of *Anopheles* mosquitoes in Benguela and Alto Catumbela

Sentinel site	Species	N^a^	Blood meal origin, n (%)							HBI
			Human	Cow	Human/cow	Dog	Goat	Pig	Unknown	
Bela Vista	*An. coluzzii*	3	2 (67)	0 (0)	0 (0)	0 (0)	0 (0)	0 (0)	1 (33)	0.67
	Total tested	3	2 (67)	0 (0)	0 (0)	0 (0)	0 (0)	0 (0)	1 (33)	0.67
Alto Catumbela	*An. arabiensis*	10	5 (50)	1 (10)	0 (0)	0 (0)	0 (0)	0 (0)	4 (40)	0.50
	*An. gambiae *s.s.	8	2 (25)	0 (0)	0 (0)	0 (0)	0 (0)	0 (0)	6 (75)	0.25
	*An. funestus *s.s.	77	61 (79)	0 (0)	4 (5)	0 (0)	0 (0)	0 (0)	12 (16)	0.84
	*An. vaneedeni*	2	1 (50)	1 (50)	0 (0)	0 (0)	0 (0)	0 (0)	0 (0)	0.50
	Total tested	97	69 (71)	2 (2)	4 (4)	0 (0)	0 (0)	0 (0)	22 (23)	0.75

^a^Number of *Anopheles* mosquitoes screened for the origin of the blood meal

### *Plasmodium falciparum* infection rate

A total of 371 female anopheline mosquitoes were screened for the presence of *P. falciparum* DNA (Table 7). Infection rate varied depending on the species and season of collections. No *P. falciparum* DNA was found in *An. arabiensis*. In the Bela Vista sentinel site, *An. coluzzii* was the only species screened with an overall infection rate of 23.1%. Overall, at Alto Catumbela, *An. funestus* s.s. had a higher *P. falciparum* infection rate of 5.7% compared to the 2.4% rate in *An. gambiae* s.s. Despite these differences, statistical analysis revealed no significant differences between the two rates (Fisher’s exact test, *P* > 0.05). When comparing the infection rate within the rainy and dry season in Alto Catumbela for *An. funestus* group and *An. gambiae* complex, no significant differences were observed between the two (Fisher’s exact test, *P* > 0.05).

**Table 7:** Monthly *Plasmodium falciparum* infection rates in anopheline mosquitoes in the two adult collection sites: Bela Vista and Alto Catumbela

Season	Month of sampling	Bela Vista			Alto Catumbela								
		*Anopheles coluzzii*			*Anopheles funestus *s.s.			*Anopheles gambiae *s.s.			*Anopheles arabiensis*		
		DNA +	N	IR %	DNA +	N	IR %	DNA +	N	IR %	DNA +	N	IR %
Dry	May	0	0	–	2	47	4.3	0	7	0.0	0	13	0.0
	Jun	1	1	100.0	–	0	–	0	1	0.0	0	0	–
	Total	1	1	100.0	2	47	4.3	0	8	0.0	0	13	0.0
Rainy	February	2	10	20.0	6	94	6.4	1	17	5.9	0	2	0.0
	March	0	0	–	9	112	8.0	0	11	0.0	0	0	0.0
	April	0	2	0.0	0	47	0.0	0	6	0.0	0	1	0.0
	Total	2	12	16.7	15	253	5.9	1	34	2.9	0	3	0.0
Total		3	13	23.1	17	300	5.7	1	42	2.4	0	16	0.0

### Knockdown resistance mutations in *Anopheles gambiae* s.l.

In Bela Vista, a total of 71 *An. coluzzii* mosquitoes were analysed for the presence of L1014F and L1014S mutations; among these, 16 were not exposed to insecticides. The genotypic frequency of L1014F in these mosquitoes showed a dominance of the resistant allele genotype, with a frequency of 0.94. In addition, the allele frequencies of L1014F in susceptible or resistant mosquitoes were similar (Fisher exact test, *P* > 0.05). When considering both phenotypes and unexposed mosquitoes, the frequency of the mutant allele was 0.71. At Alto Catumbela site, 76 mosquitoes, comprising *An. arabiensis* and *An. gambiae* s.s., were analysed. The *An. arabiensis* group, consisting of 26 mosquitoes, did not exhibit the L1014F mutation, whereas the *An. gambiae* s.s., with 49 mosquitoes, predominantly exhibited the mutant allele with an allele frequency of 0.90. Allele 1014S was reported for the first time to our knowledge in *An. gambiae* s.s. from the province of Benguela, only in heterozygosity with the 1014F allele. In total, across both sentinel sites, 148 mosquitoes were analysed. The overall frequency of 1014F mutation was 0.65, while the 1014S mutation was found to be 0.01 (Table 8).

**Table 8:** Genotype frequencies of L1014F and L1014S in *Anopheles* spp. from a member of *An. gambiae* complex from Benguela Province

Sentinel site	Species	Phenotype	N	Genotype frequency of L1014F			Genotype frequency of L1014S			F (Phe)	F (Ser)
				Leu/Leu	Leu/Phe	Phe/Phe	Leu/Ser	Phe/Ser	Ser/Ser		
Bela Vista	*An. coluzzii*	NA	16	0	2	14	0	0	0	0.94	0.00
		Resistant	25	4	12	9	0	0	0	0.60	0.00
		Susceptible	30	3	13	14	0	0	0	0.68	0.00
		Sub-total	71	7	27	37	0	0	0	0.71	0.00
	Hybrid	Susceptible	1	0	1	0	0	0	0	0.50	0.00
	Subtotal		72	14	28	37	0	0	0	0.71	0.00
Alto Catumbela	*An. arabiensis*	NA	26	26	0	0	0	0	0	0.00	0.00
	*An. gambiae s.s*	NA	49	0	4	42	0	3	0	0.90	0.03
	Hybrid	NA	1	0	1	0	0	0	0	0.50	0.00
	Subtotal		76	26	5	42	0	3	0	0.59	0.02
Total			148	33	33	79	0	3	0	0.65	0.01

NA: not applicable, mosquitoes caught as adults and not exposed to insecticides

## Discussion

This study significantly advances our understanding of the *Anopheles* spp. populations in urban and rural settings in Benguela Province, highlighting key aspects of malaria transmission. Our research confirms the presence of *An. coluzzii*, *An. gambiae* s.s., *An. arabiensis*, and *An. funestus* s.s., consistent with prior studies in Angola and sub-Saharan Africa [2, 5–7]. The relatively low number of *An. gambiae* complex members collected indoors may suggest a behavioural change due to ITN presence as previously reported in other locations [30, 31]. We confirm the presence of *An. coustani* s.l., *An. squamosus*, *An. pretoriensis*, and the first report to our knowledge of *An. vaneedeni* in Alto Catumbela sentinel site. These species were found infected with *P. falciparum* in other sub-Saharan Africa countries [32–35]. These results highlight the importance of continued investigation into the roles that less studied species play in the transmission of malaria in the province. *Anopheles coluzzii/An. gambiae* s.s. hybrids were reported from Cavaco River and Alto Catumbela sentinel site during the dry season. The use of molecular methods revealed a significant accuracy in morphological identification done in the field, especially for the *An. funestus* group (97.3%) and *An. gambiae* complex (98.1%), the primary malaria vectors in Angola. This emphasises the importance of skilled technical staff and robust surveillance networks in malaria control strategies. In Bela Vista, we observed an overall *P. falciparum* infection rate of 23.1% in *An. coluzzii*, surpassing the 1.9% rate reported by Cuamba et al. [5]. This discrepancy may not be solely attributed to variations in sample sizes or collection methods. Environmental factors, such as the region’s unique ecological and climatic conditions, may enhance breeding and survival rates of *Anopheles* mosquitoes, particularly *An. coluzzii* and *An. funestus* s.s. Additionally, changes in human behaviour and vector control measures, alongside potential genetic variations in *Plasmodium* strains and varying immunity levels within the human population, could contribute to the observed high infection rates. The consistency of *P. falciparum* infection rates among *An. funestus* s.s. populations across seasons further underscores the species’ role in indoor malaria transmission within the Alto Catumbela region, highlighting the complex interplay of factors influencing malaria dynamics. This observation aligns with findings from other provinces, further emphasising its role in malaria transmission [6, 36]. Our study’s novel discovery of the West African kdr-resistance allele 1014F in *An. coluzzii* and *An. gambiae* s.s. confirms the presence of pyrethroid resistance in these populations. Interestingly, the first detection to our knowledge of the East African kdr-resistance allele 1014S in *An. gambiae* s.s. highlights the emergence of this mutation in Benguela Province, showing the importance of continuous monitoring of the setting of insecticide resistances in *Anopheles gambiae* s.s. populations. This finding suggests an emerging challenge in insecticide resistance, previously unrecorded in this region [5, 6, 26, 37]. Multiple factors might be responsible for this resistance, including the selective pressure exerted by ITN. The absence of kdr mutations in *An. arabiensis* indicates potential susceptibility to pyrethroids, warranting further investigation. However, our study has limitations. The geographic scope, limited to two sentinel sites, and the method of collection might not fully represent the broader *Anopheles* population dynamics. Future research should explore genetic diversity and resistance patterns more comprehensively. The detection of mutations involved in pyrethroid resistance was limited to L1014F and L1014S in *An. gambiae s.l.* Other potential mechanisms of insecticide resistance, such as metabolic resistance, were not yet explored in Angola. Future studies should investigate additional resistance mechanisms to provide a more comprehensive understanding of insecticide resistance across malaria vector populations. Additionally, a deeper investigation into the vectors’ feeding behaviours is necessary for a complete understanding of malaria transmission dynamics.

## Conclusion

In conclusion, our findings provide crucial insights into the malaria vector populations in Angola, with significant implications for public health policy, vital for tailoring vector control strategies, ensuring their continued efficacy. This study not only fills a critical knowledge gap but also lays the groundwork for enhancing malaria entomological surveillance and control efforts in Angola.

## Data Availability

The data and materials that support the findings of this study are available from the corresponding author upon request. Sequences have been submitted to NCBI Genbank database.

## Abbreviations

COI: Cytochrome c oxidase subunit
PCR: Polymerase chain reaction
HBI: Human blood index
ITN: Insecticide-treated nets
KDR: Knockdown resistance
IRS: Indoor residual spraying
